# Telephone consultations in place of face to face out-patient consultations for patients discharged from hospital following surgery: a systematic review

**DOI:** 10.1186/1472-6963-13-128

**Published:** 2013-04-05

**Authors:** Jo Thompson-Coon, Abdul-Kareem Abdul-Rahman, Rebecca Whear, Alison Bethel, Bijay Vaidya, Christian A Gericke, Ken Stein

**Affiliations:** 1 PenCLAHRC, University of Exeter Medical School, University of Exeter, Veysey Building, Salmon Pool Lane, Exeter, EX2 4SF, UK; 2 Department of Endocrinology, Royal Devon & Exeter Hospital NHS Foundation Trust, Exeter, EX2 5DW, UK; 3 PenCLAHRC, National Institute for Health Research, Peninsula College of Medicine and Dentistry, University of Plymouth, Portland Square, Plymouth, PL4 8AA, UK; 4 The Wesley Research Institute and Queenland University of Technology, Brisbane, QLD, 4068, Australia

**Keywords:** Systematic review, Outpatient appointment scheduling, Surgery, Routine follow-up, Telephone consultation

## Abstract

**Background:**

Routine follow-up following uncomplicated surgery is being delivered by telephone in some settings. Telephone consultations may be preferable to patients and improve outpatient resource use. We aimed to compare the effectiveness of telephone consultations with face to face follow-up consultations, in patients discharged from hospital following surgery.

**Methods:**

Seven electronic databases (including Medline, Embase and PsycINFO) were searched from inception to July 2011. Comparative studies of any design in which routine follow-up via telephone was compared with face to face consultation in patients discharged from hospital after surgery were included. Study selection, data extraction and quality appraisal were performed independently by two reviewers with consensus reached by discussion and involvement of a third reviewer where necessary.

**Results:**

Five papers (four studies; 865 adults) met the inclusion criteria. The studies were of low methodological quality and reported dissimilar outcomes precluding any formal synthesis.

**Conclusions:**

There has been very little comparative evaluation of different methods of routine follow-up care in patients discharged from hospital following surgery. Further work is needed to establish a role for telephone consultation in this patient group.

## Background

Rising costs and increasing demand for specialist services have increased interest in maximising the efficiency of managing specialist outpatient departments [1-8]. In this context, telephone follow-up consultations for various medical and surgical patient populations have been suggested as a means of improving the use of outpatient resources [9-12].

Providing routine follow-up by telephone may be considered preferable in situations where patients have to travel from rural areas, and for those whose health or social conditions make hospital visits difficult [13]. Telephone consultations may also allow valuable outpatient clinic time to be occupied by those patients who have a more severe or complex conditions. This form of follow-up might also help improve compliance with scheduled follow-ups, avert the need for health care professionals to provide home visits, and prevent unnecessary return visits to the emergency department [3,14].

However, it is also possible that telephone follow-up consultations may become an additional burden to the outpatient department, instead of reducing staff workloads with clinicians wary of missing or being unable to confirm important clinical signs or other disease conditions [9,15,16].

Anecdotal evidence suggests that surgical teams are routinely replacing face to face follow-up consultations with those made by telephone in some patient groups, where the intention is to discharge the patient from surgical specialist care. To date, there has been no synthesis of the evidence for the direct comparison between face to face follow-up consultations and those made by telephone in the context of surgical follow up care.

We therefore performed a systematic review to assess the effectiveness and, if possible, the cost effectiveness of telephone consultation as a method for routine follow-up consultations in secondary care for those patients who have undergone a surgical procedure, in comparison to (or as a replacement for) conducting follow-up appointments face to face.

## Methods

The systematic review was conducted following the general principles published by the NHS Centre for Reviews and Dissemination (CRD) [17]. A pre-defined protocol was developed following consultation with topic and methods experts and is available from the PenCLAHRC website [http://clahrc-peninsula.nihr.ac.uk/est-projects.php].

### Literature search and eligibility criteria

The search strategy was developed after consultation with clinical experts (RF, CG, BV) and examination of key papers. The search strategy for PsycINFO is shown below; the search was adapted to run in other databases. Studies were identified by searching the following electronic databases: Medline, Embase and PsycINFO (all via Ovid); AMED and CINAHL (both via NHS Evidence); and the Cochrane Library; and the Social Sciences Citation Index (in the Web of Science). These databases were searched from inception until July 2011. No search filters were applied. Due to resource constraints, only articles published in English were included. Reference lists of included papers were checked for further potentially relevant citations. The search was updated in June 2012.

Search strategy for PsycINFO (OVID)

Database: psycINFO <1806 to July Week 2 2012>

Search strategy:

1. exp surgery/ (33266)

2. exp Outpatients/ (4582)

3. exp telephone systems/ (2253)

4. exp clinics/ (4514)

5. exp Aftercare/ (912)

6. exp Hospital Discharge/ (2135)

7. 4 or 5 or 6 (7437)

8. 1 or 2 (37824)

9. 3 and 7 and 8 (4)

10.  (surger* or surgi* or hospital* or outpatient* or operat*).tw. (225907)

11.  (secondary adj care).tw. (476)

12.  10 or 11 (226244)

13.  (tele* or phone* or call? or calling).tw. (81736)

14.  (appointment* or consult* or clinic* or $discharge or review* or followup or aftercare).tw. (662394)

15.  (follow adj up).tw. (63368)

16.  14 or 15 (698170)

17.  (tele* or phone* or call? or calling).ti. (16117)

18.  ((tele* or phone* or call? or calling) adj5 (appointment* or consult* or clinic* or $discharge or review* or followup or aftercare)).tw. (2301)

19.  17 and 18 (881)

20.  14 and 17 (2600)

21.  19 or 20 (2600)

22.  12 and 21 (409)

Studies were included if they compared the effects of telephone with face to face follow up consultations in patients who had undergone a surgical procedure in secondary care. Patients included in the studies had to be assessed as fit by their health care providers to be discharged to home and to receive a follow-up appointment in the surgical outpatient clinic. The telephone follow-up consultations needed to be planned and initiated by health care professionals and could be provided live or pre-recorded (or be automated). We excluded consultations in only text form. No limits on reported outcomes were imposed. Any study reporting comparative data was included irrespective of study design.

### Study selection

Three authors (AA/JTC and RW) independently screened the titles and abstracts and applied the inclusion and exclusion criteria in a standardised and blinded manner. Duplicates were identified and discrepancies were reconciled through discussion. The full text of articles that were assessed as potentially meeting the inclusion criteria at this stage were retrieved and underwent the same process. Disagreements were arbitrated and resolved by discussion with others in the team (JTC and KS).

### Data extraction

Data extraction was performed using a piloted data extraction form by one reviewer (AA/JTC or RW) and checked by a second (AA or RW). Disagreements were reconciled through discussion.

The following data were extracted for each study: 1) characteristics of the article (including author, title and year of the publication); 2) characteristics of the study participants (including mean age, underlying medical or surgical condition, the type surgical procedure performed, and inclusion and exclusion criteria); 3) characteristics of the study (such as the study design, sampling assumptions, whether the sample was derived randomly, the method of randomisation to intervention allocations, method of concealment, and method of blinding); 4) description of the intervention (who provided the telephone follow-up consultation, how soon after surgery it was carried out, and its duration); 5) descriptions of the comparator (including whether it was a face to face follow-up consultation, when it was provided, and duration); and 6) type of outcomes reported (including the variables reported, the values of the results, the standard deviations, and any statistical tests of association). The data extraction template is available from the authors on request.

### Assessment of quality and risk of bias

Risk of bias was assessed using the principles published by the NHS Centre for Reviews and Dissemination [17], and the Cochrane Handbook of Systematic Reviews for Interventions [18]. One reviewer applied the criteria (AA/JTC or RW) which were checked by a second reviewer (AA/JTC or RW). Disagreements were resolved by discussion.

### Data synthesis

Due to the heterogeneity of the study designs, the population and outcome measures reported in them, and the risk of bias identified, no formal statistical synthesis was performed. The results of the included studies were tabulated and evaluated in a narrative manner.

## Results

### Study selection

**Figure 1:**
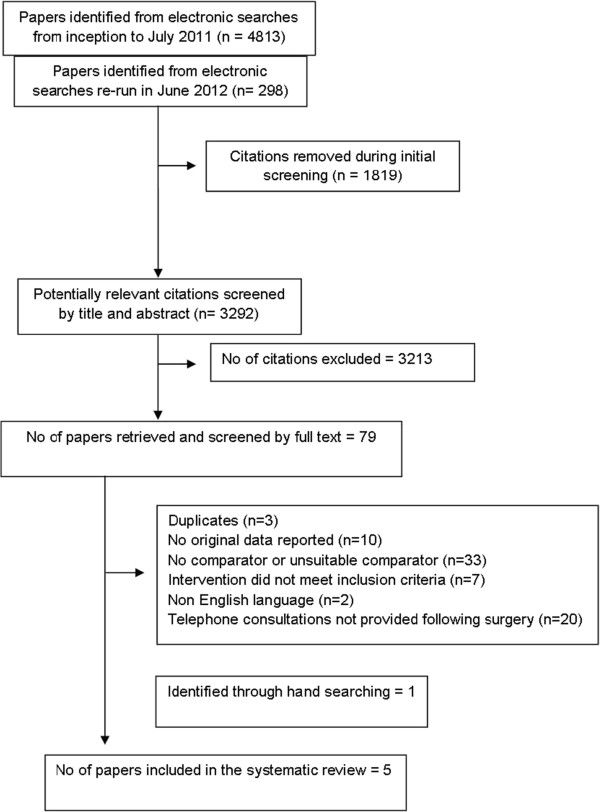
Process of study selection.

A total of 4813 articles were identified by the initial search. Of these, 2,994 were unique (non duplicate) citations. The search was re-run in June 2012 and identified a further 298 unique references. Title and abstract screening resulted in the exclusion of 3213 citations. The full text of the remaining 79 articles was retrieved for further assessment. Seventy-five papers were excluded; three articles were duplicates, 10 were non empirical works, 33 did not include a comparator or the comparator was not face to face consultation in the outpatient department, in seven papers the intervention did not meet the inclusion criteria for the review and two papers were published in languages other than English. In 20 studies, the follow-up consultations were not provided directly following or in relation to surgery. One additional study was identified through hand searching. Five papers (four studies) were included in the review [19-23]. The process of study selection is summarised in Figure 1.

### Characteristics of included studies

Two publications were concerned with post-operative care following dental work in maxillofacial surgery departments in the US; one a retrospective audit of patient records during a time period in which both telephone consultation and traditional outpatient follow-up strategies were in use [22] and the other an RCT in which patients were randomised to either method of follow-up [21]. One paper reports prospective data collected in a non-randomised trial on the post-operative experience following cataract surgery [23]. The other two publications report the findings of a single study in people undergoing nasal septal surgery in an otorhinolaryngology department in the UK [19,20]. The data for those undergoing telephone consultation follow-up in this study was collected prospectively and compared with historical records of patients seen traditionally in the outpatient department. In all studies the telephone consultation was standardised by the use of a proforma or questionnaire and was conducted at the same time point as the comparative clinic appointment. The characteristics of the included studies are summarised in Table 1. Sample size ranged from 48 in the RCT [21] to 364 in the retrospective audit of patient notes [22]; no sample size estimations were reported. Overall, the methodological quality of the included studies was poor with a moderate to high risk of bias for all results. Although described as a randomised trial, Sittitavornwong and colleagues [21] did not report any details of the process of randomisation or allocation concealment and the reporting of the results is very unclear. It is not clear how individuals were allocated to telephone or face to face follow-up in the studies by Susarla and colleagues [22] and Mandal and colleagues [23]. Retrospective sampling of patients over different time periods may subject the results to confounding influences which are not fully evident or reported. The methodological quality of each study is summarised in Table 2.

**Table 1:** Study characteristics

**Study, year**	**Design**	**Setting**	**Population**	**Data collection periods**	**n**	**Intervention / comparator**	**Outcomes measured**
Uppal, 2003 [19] and Uppal, 2004 [20]	Historically controlled trial	Otolaryngology department; UK hospital	Patients undergoing nasal septal surgery	Dec 2000 to Jan 2002	75	Telephone call with ENT nurse six weeks after surgery using standardised protocol	Patient satisfaction with follow-up
							Direct costs
							Indirect costs
							Total costs of follow-up
			mean age 42 years; 76% male	Jan 1999 to Dec 2000	78	Clinic appointment with surgeon (time not specified)	
Sittitavornwong, 2005 [21]	RCT	Oral and maxillofacial surgery department, US University	Patients undergoing third molar removal performed under general anaesthetic	Not reported	23	Telephone call at two weeks after surgery using a questionnaire	Patient satisfaction with follow-up
							Post-operative morbidity
			mean age 20 years; 38% male		25	Clinic appointment at two weeks	Incidence of post-operative help
Susarla, 2011 [22]	Retrospective cohort	Oral and maxillofacial surgery department in a tertiary referral centre, US	Patients undergoing tooth extraction in an ambulatory setting	July 2007 to June 2009	155	Telephone call with surgeon five to 10 days after surgery using a standardised proforma	Frequency of intra-operative or postoperative complication
							Compliance with follow-up
							Patient satisfaction with follow-up
			mean age 28.6 years; 39% male		209	Clinic appointment with surgeon seven to 10 days after surgery	No. of post-operative visits
Mandal, 2004 [23]	Prospective cohort	Eye infirmary; UK hospital	Patients undergoing cataract surgery	Not reported	100	Home visit by ophthalmic nurse one day after surgery	Feelings of reassurance
							Level of understanding of post-operative information
					100	Clinic appointment with ophthalmic nurse one day after surgery	Satisfaction with length of review
					100	Telephone call using a structured review form one day after surgery	

**Table 2:** Indicators of study quality

**Quality indicator**	**Uppal, 2003**[19]**; Uppal, 2004**[20]	**Sittitavornwong, 2005**[21]	**Susarla, 2011**[22]	**Mandal, 2004**[23]
Was the objective of the study clearly described?	Yes	Yes	Yes	Yes
Are the main outcomes to be measured clearly described?	Yes	Yes	Yes	Yes
Are the characteristics of the included subjects clearly described?	Yes	No	Partial	No
Was there a clearly defined point in time when the intervention occurred?	Yes	Yes	Yes	No
Are the interventions of interest clearly described?	Yes	No	Yes	Yes
Are possible confounders clearly described?	Partial	Partial	Partial	No
Are the main findings of the study clearly described?	Yes	Yes	Yes	Yes
Are estimates of the random variability in the data provided?	Yes	Yes	Yes	No
Are all adverse events that may be a consequence of the intervention reported?	Unclear	Unclear	Unclear	Unclear
Are the characteristics of patients lost to follow-up described?	NA	Yes	NA	No
Were losses of patients to follow up accounted for?	NA	Yes	NA	No
Are the actual probability values reported for the main outcomes?	Yes	No	Yes	No
Were attempts made to blind outcome assessors to the intervention?	NA	No	NA	NA
Were the groups recruited from the same population?	No	Yes	Yes	Yes
Were the groups recruited over the same period of time?	No	Yes	Yes	Unclear
Was a representative sample of the population approached?	Yes	Yes	Unclear	Unclear
Was the intervention unlikely to affect data collection?	Yes	Yes	Yes	Yes
Was there reliable compliance with the intervention?	Partial	Yes	NA	Yes
Were the main outcome measures used valid and reliable?	Yes	Yes	Yes	No
Was the study free from selective outcome reporting?	Unclear	Unclear	Unclear	Unclear
Were baseline characteristics similar between groups?	Unclear	Unclear	Unclear	Unclear

### Patient satisfaction outcomes

Patient satisfaction has been measured in a number of ways across these four studies, precluding a simple summary of the findings. However, the results seem to suggest that patients were happy to receive routine follow-up care by telephone and in some cases showed a preference for this method. It is possible that the method used to administer the questionnaires e.g. face to face or over the telephone might have influenced the results; we were unable to look at this in detail due to the small number of included studies. In the study by Uppal and colleagues [19], a modified version of a validated outpatient satisfaction questionnaire was used; the language was modified slightly so that the same questionnaire could be used with both groups of patients. Patients were asked to respond on a five-point Likert scale (ranging from strongly agree to strongly disagree). The questions addressed issues such as whether the individual conducting the review was courteous and helpful and whether they listened to the patient. A total score was calculated for each patient and the results indicate that patient satisfaction was greater with nurse-led telephone follow-up than with conventional outpatient follow-up (mean difference in scores 0.39; 95% CI 0.17 to 0.61; p = 0.001). Mandal and colleagues [23] used a non-validated questionnaire designed to assess the acceptability of telephone review as a post-operative assessment technique. The questions focussed on reassurance, comprehension and satisfaction with the service provided. There was no statistically significant difference in the proportion of patients saying that they were ‘very reassured’ or ‘reassured’ in the telephone review group compared with the eye infirmary clinic (although the difference between telephone review and review in the home visit group was significant). There were no significant differences in the level of understanding of post-operative materials between groups, nor in the assessment of the length of follow-up with all groups having a good level of comprehension and feeling that the length of the review was ‘just right’. Although patients were asked which method of follow-up they preferred, their responses were not independent of their allocated group. More than 90% of patients in the home visit group indicated a preference for a home visit and more than 70% of the telephone group said that they preferred telephone follow-up. Sittitavornwong and colleagues [21] report that 73% (35 of 48 patients) preferred telephone follow-up and 27% (13 of 48) preferred clinic follow-up. These results are difficult to interpret since they provide information on the proportion of the total number of patients in the study (n = 48) reporting these preferences, rather than the proportion per randomised group. Susarla and colleagues asked patients if they were satisfied with the telephone post-operative follow-up compared with a clinic visit and the answer was recorded as ‘yes’ or ‘no’ [22].

### Costs

Two studies report costs [20,23]. Uppal and colleagues in 2004 estimate the direct and indirect health care related costs of telephone follow-up consultations at £50 per patient, compared to £106.11 for those conducted face to face [20]. A simple cost calculation in the paper by Mandal and colleagues estimates the cost of a telephone review at between £1 and £2; it is not clear which costs have been included in these calculations and there is no information provided about the cost of an outpatient appointment in the eye infirmary (the estimated cost of a home visit was £8.50) [23].

### Frequency of complications

The data provided by Sittitavornwong and colleagues is difficult to interpret since they provide information on the proportion of the total number of patients in the study experiencing complications rather than the number per randomised group [21] (Table 3). In the study by Susarla and colleagues [22], there were significantly more complications in the group receiving telephone review. However, when the results were adjusted to account for differences in the two groups, no statistically significant difference was seen.

**Table 3:** Study results

**Study, year**	**Uppal, 2003**[19]**; Uppal, 2004**[20]		**Sittitavornwong, 2005**[21]		**Susarla, 2011**[22]		**Mandal, 2004**[23]		
	Telephone	Face to face	Telephone	Face to face	Telephone	Face to face	Telephone	Home visit	Face to Face
n	75	75	25	23	155	209	100^$^	100^$^	100^$^
**Cost outcomes**									
Total cost of follow-up (£)	3760.88	7958.25	-	-	-	-	-	-	-
Cost per patient (£)	50.15	106.11	-	-	-	-	1 to 2	8.50	-
**Patient satisfaction outcomes**									
Patient satisfaction with follow-up	1.04 (0.50)*^§^	0.65 (0.52) *^§^	-	-	95.9%^†^	-	-	-	-
Proportion reporting ‘very reassured’	-	-	-	-	-	-	69.6%	83.9%	79.5%
Proportion showing good level of understanding	-	-	-	-	-	-	84%	93.1%	87.2%
Patient preference for follow-up method	-	-	73%**	27%**			-	-	-
Satisfaction with length of review	-	-	-	-	-	-	97.2% just right		
Compliance with follow-up	83%	93%	83%	77%	97.1%	94.5%	-	-	-
**Frequency of complications**									
Frequency of intra-operative or post-operative complication	-	-			12.9%^#^	23.4%^#^	-	-	-
on day one			19% (n = 9)**	10% (n = 5)**					
at two weeks			10% (n = 5)**	2% (n = 1)**					
Incidence of post-operative help	-	-	8% (n = 4)**	6% (n = 3)**			-	-	-

*mean (sd); ^§^missing data for 33 patients in the telephone group and 29 patients in the face to face group; ** these data are reported as a proportion of 48 patients;

^†^ self reported patient satisfaction rate in 82% of patients; ^#^ after adjusting for differences between the samples, no significant difference was found in complication frequencies (p = 0.7); ^$^only 82% of patients completed or partially completed questionnaires; - not reported.

## Discussion

Despite conducting a widespread search for possible data in a variety of sources we were unable to identify any good quality comparative evidence for the use of telephone consultations in place of face to face outpatient appointments after surgery. Although from a limited pool of surgical modalities, data from all four studies suggests that patients were happy to receive routine follow-up care by telephone and in most instances showed some preference to this method compared with an appointment in the outpatient department.

However, the methodological quality of all the identified studies was poor with a moderate to high risk of bias for all the results. In addition, the studies were small, patient satisfaction was measured in a variety of ways and there is little information available on long-term outcomes. The limitations of the included studies have consequences for any sensible extrapolation of the results to other clinical situations.

This research question arose following discussion with surgeons in our local hospital who are routinely replacing face to face outpatient appointments with telephone calls following some surgical procedures. In an environment of increasing cost and demand and a need for ever greater efficiency, it is disappointing that we were able to identify so little comparative research.

In this review, we have taken considerable steps to identify all relevant published and unpublished papers by searching seven electronic databases and hand searching the reference lists of all included papers and many others identified during the search. Although we were only able to include papers published in the English language due to resource constraint, which may have resulted in the omission of some evidence, our searches were not limited to English only and did not identify a significant body of literature in other languages. Very few studies have compared the methods of care and as far as we are aware, this is the first systematic review to specifically address the effectiveness and acceptability of telephone consultation in place of face to face outpatient appointments in patients following uncomplicated surgery.

The authors of a Cochrane Review of telephone consultations in the three months following discharge from hospital identified 33 papers in which telephone follow-up consultations had been evaluated following a wide range of treatments in many patient groups, using a range of comparators and using a variety of outcome measures [24]. In most cases there was no statistically significant difference between telephone and control groups and no meta-analysis was possible. None of the studies identified adverse effects of telephone follow-up. Our review takes a more focused approach than the previous work, addressing explicitly the use of telephone follow up after surgery and compared this with face-to-face hospital based appointments (not home visits). None of the studies included in our review were identified in the Cochrane Review. Furthermore, we have looked at complications related to the surgical operation as this goes to the issue of whether telephone consultations in this context are safe – a necessary pre-requisite for their implementation as routine practice.

Post-surgical consultations may be used for the exchange of a variety of different kinds of information e.g. education, identification of complications, reassurance, management of pain and symptoms. Moreover, the aim for consultations, in most cases, will be discharge from specialist care. It is therefore necessary that the consultation takes careful account of all possible harms and patient concerns. Telephone consultation have been studied in only a small number of conditions and, while they appear to be an innovative use of health service resources, uncertainty remains about their effects and whether these are consistent in different conditions.

There is therefore a need for good quality prospective and, crucially, comparative evaluations of telephone consultations with outpatient appointments in post-surgical care which seek to collect information on all aspects of care. Whilst telephone consultations might be more convenient for patients, there is no indication from the published literature whether this type of after-care has any impact on the frequency of adverse events.

## Conclusion

There has been very little comparative evaluation of different methods of routine follow-up care in patients discharged from hospital following surgery. Further rigorous work is needed to establish a role for telephone consultation in this patient group as a replacement for face to face follow up in hospital outpatient departments.

## Competing interests

The authors declare that they have no competing interests.

## Authors’ contributions

JTC was substantially involved in the conception and design of this review as well as data analysis and interpretation. JTC was also involved in writing and revising the manuscript and has approved this final version. AA was substantially involved in the conception and design of this review as well as data analysis and interpretation. AA was also involved in writing the manuscript and has approved this final version. RW was substantially involved in the conception and design of this review as well as data analysis and interpretation. RW was also involved in revising the manuscript and has approved this final version. AB was substantially involved in running the literature searches, managing the databases and retrieving articles. AB was also involved in revising the manuscript and has approved this final version. BV was substantially involved in the conception and design of this review. BV was also involved in revising the manuscript and has approved this final version. CG was involved in the conception and design of this review and was also involved in revising the manuscript and has approved this final version. KS was substantially involved in the conception and design of this review as well as its interpretation. KS was also involved in revising the manuscript and has approved this final version. All authors read and approved the final manuscript.

## Pre-publication history

The pre-publication history for this paper can be accessed here:

http://www.biomedcentral.com/1472-6963/13/128/prepub
